# Pigmented Purpuric Dermatosis Following Tumor Necrosis Factor-Alpha Inhibitor Therapy: A Case Report

**DOI:** 10.5152/ArchRheumatol.2025.11094

**Published:** 2025-06-23

**Authors:** Sinem Kübra Beke, Hüseyin Kaplan, Gizem Cengiz, Kemal Deniz, Demet Kartal

**Affiliations:** 1 Department of Physical Medicine and Rehabilitation, Division of Rheumatology, Erciyes University Faculty of Medicine, Kayseri, Türkiye; 2 Department of Pathology, Erciyes University Faculty of Medicine, Kayseri, Türkiye; 3 Department of Dermatology and Venereology, Erciyes University Faculty of Medicine, Kayseri, Türkiye

Dear Editor,

Pigmented purpuric dermatoses (PPD) are chronic skin diseases with recurrent lesions and similar histopathological features, including perivascular lymphocytic inflammation, epidermal changes, and erythrocyte extravasation.^1^ Tumor necrosis factor-alpha (TNF-α) inhibitors are widely used in rheumatological disease management and have been associated with several skin reactions; however, reports of PPD are rare.^2^ This article presents a case of PPD triggered by TNF-α inhibitor therapy.

A 60-year-old female with seropositive rheumatoid arthritis on methotrexate, leflunomide, sulfasalazine, and prednisolone presented to the clinic with worsening symptoms despite combined conventional synthetic disease-modifying antirheumatic drug (DMARD) therapy. Her history included total thyroidectomy and hypertension. Examination revealed 4 tender and 2 swollen joints, with no other abnormalities. Laboratory tests showed C-reactive protein was 19.64 mg/L, and erythrocyte sedimentation rate was 31 mm/hour. Due to high disease activity (Disease Activity Score 28: 5.97), etanercept was started. In the second month of etanercept treatment, the patient developed rashes on both lower extremities (Figure 1A). Physical examination and all laboratory tests, including complete blood count, biochemical tests, autoantibody profiles, complement levels, and viral serologies (hepatitis and HIV), were normal. A dermatology consultation was obtained, and a skin biopsy was performed, revealing findings consistent with PPD (Figure 2). Topical corticosteroids provided partial relief but did not fully resolve the rashes. Given the temporal relationship between etanercept and the development of rashes, the drug was stopped, and adalimumab was started. Therapy was discontinued due to continued joint pain and the persistence of rashes while on adalimumab, and an interleukin-6 inhibitor, tocilizumab, was initiated. At the 1-month follow-up after starting tocilizumab, a notable improvement in rashes was documented (Figure 1). Long-term follow-up is ongoing. Informed consent for publication was obtained from the patient.

The etiology of PPD is not fully understood, but its pathogenesis is thought to be driven by cell-mediated immune mechanisms.^1^ Drugs such as statins, beta-blockers, calcium channel blockers, aspirin, and diuretics have been associated with PPD.^3,4^ The strength of the association between the drug and the adverse reaction was evaluated using the Naranjo Adverse Drug Reaction Probability Scale. Scores ≥5 to 8 on this scale indicate a “probable” relationship, while scores ≥9 indicate a “definite” relationship. A Naranjo score of 6 confirmed a probable association between the rash and TNF-α inhibitors.^5^ It is a rare disease, having been reported in just 2 cases associated with the use of TNF-α inhibitors.^6,7^ In one of these 2 cases, the trigger was promoted by non-TNF, while in the other case, the trigger was TNF, and with the discontinuation of the drug, the lesions were resolved.^8^ The persistence of rashes in this case parallels findings from previously reported cases, emphasizing the potential class effect of TNF-α inhibitors. PPD should be distinguished from vasculitis and similar conditions, and a skin biopsy serves as a valuable diagnostic tool.^4^ In this case, at the 1-month follow-up visit at which the tocilizumab therapy was initiated, rashes almost completely regressed (Figure 1B), and the follow-up process is still ongoing. This case highlights the importance of careful monitoring for skin reactions in patients receiving TNF-α inhibitors and emphasizes the need for a multidisciplinary approach to ensure optimal management.

## Figures and Tables

**Figure 1.**
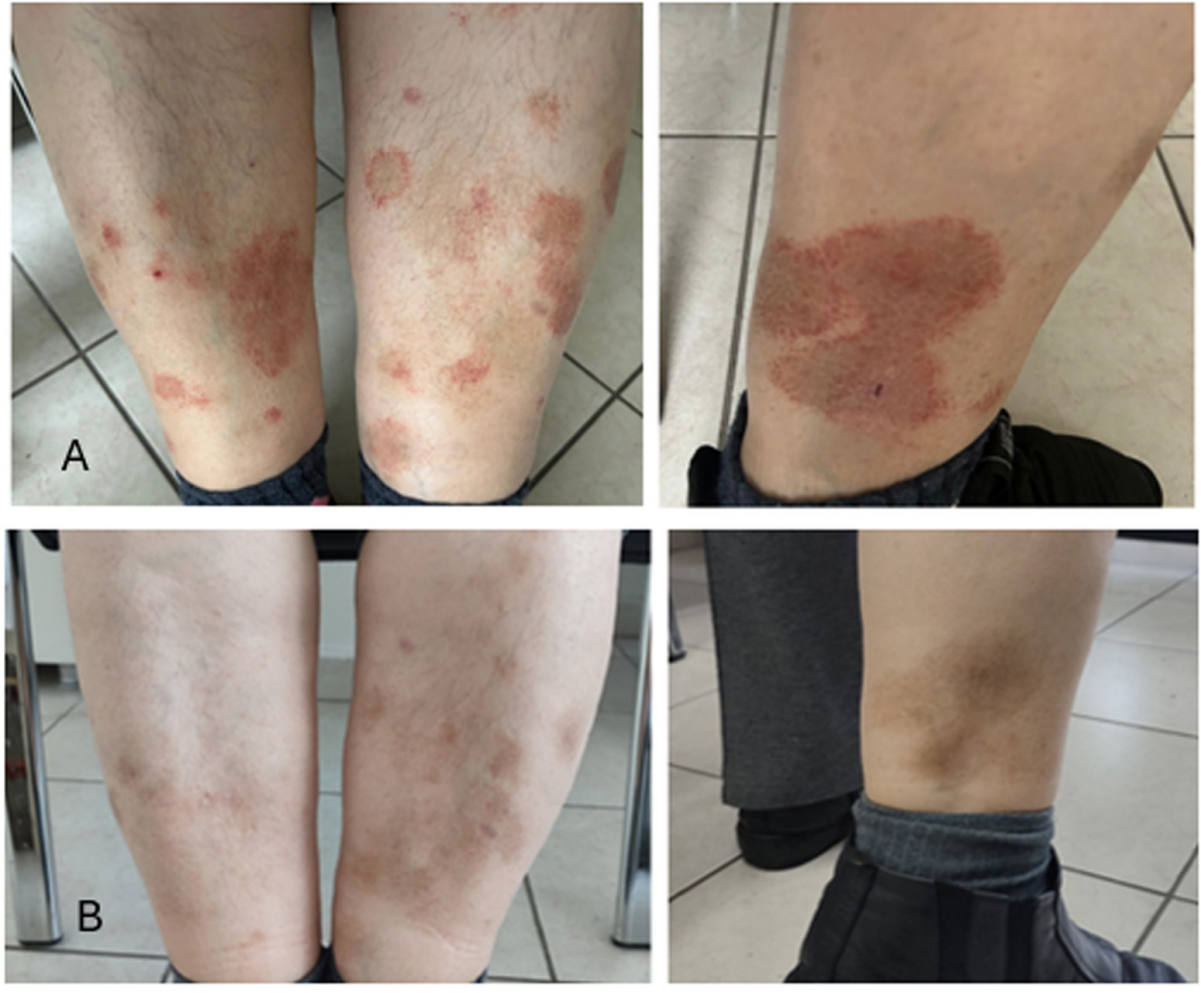
(A) Rash on the lower extremities during etanercept treatment, and (B) resolution of the rash after one month of tocilizumab therapy.

**Figure 2.**
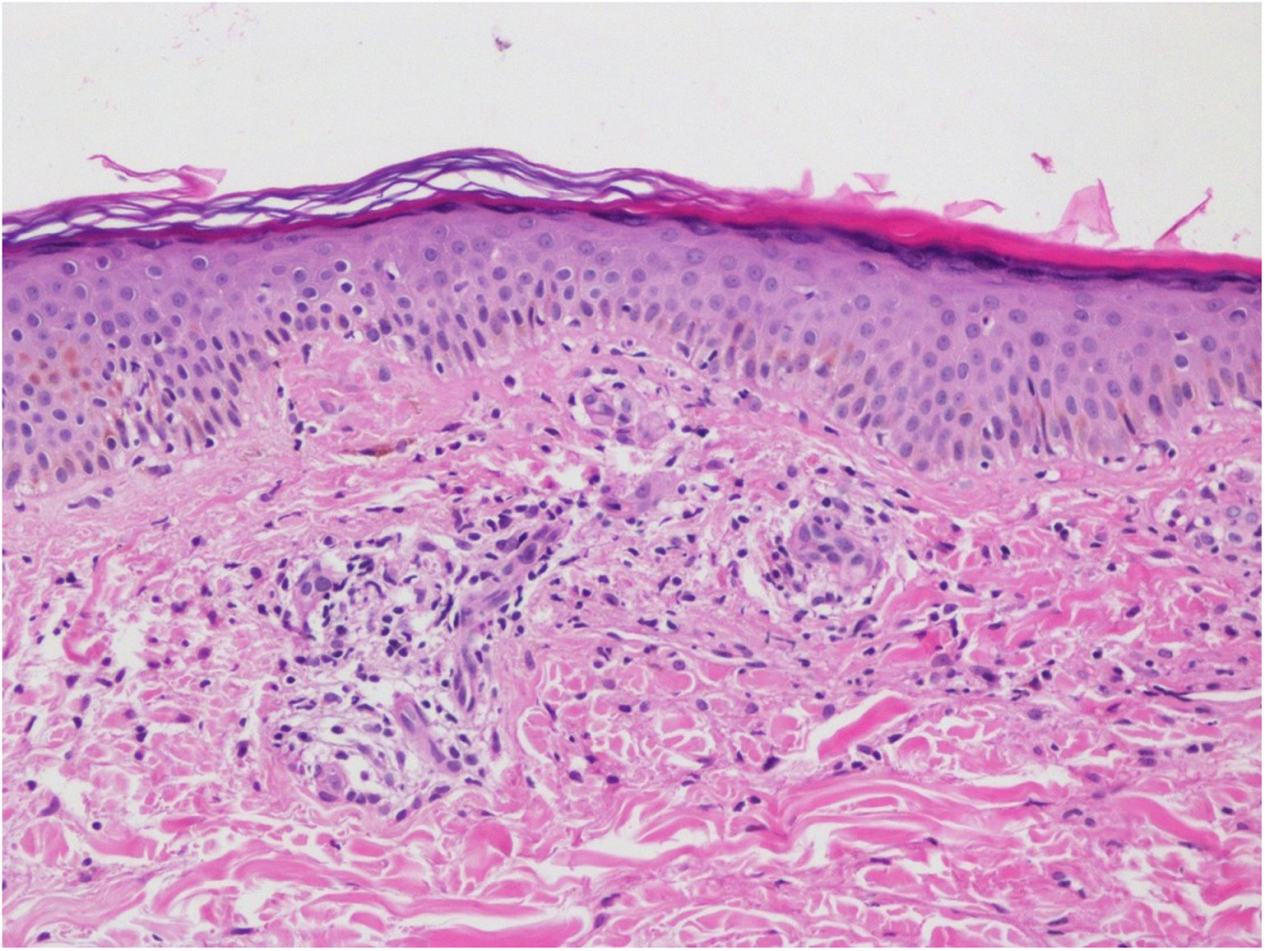
In the papillary dermis, vascular endothelial swelling, perivascular lymphocytic infiltration, and pigmented macrophages were observed.

## Data Availability

The data that support the findings of this study are available on request from the corresponding author.
